# Gloss discrimination: Toward an image-based perceptual model

**DOI:** 10.1167/jov.25.10.6

**Published:** 2025-08-11

**Authors:** Jacob R. Cheeseman, James A. Ferwerda, Takuma Morimoto, Roland W. Fleming

**Affiliations:** 1 Department of Experimental Psychology, Justus Liebig University Giessen, Giessen, Germany; 2 Carlson Center for Imaging Science, Rochester Institute of Technology, Rochester, NY, USA; 3 Physics Center of Minho and Porto Universities (CF-UM-UP), Braga, Portugal; 4 Department of Experimental Psychology, University of Oxford, Oxford, UK; 5 Center for Mind, Brain and Behavior (CMBB), Marburg, Germany

**Keywords:** gloss, material perception, surface reflectance, thresholds, JNDs

## Abstract

Gloss is typically considered the perceptual counterpart of a surface's reflectance characteristics. Yet, asking how discriminable two surfaces are on the basis of surface properties is a poorly posed question, as scene factors other than reflectance can have substantial effects on how discriminable two glossy surfaces are to humans. This difficulty with predicting gloss discrimination has so far hobbled efforts to establish a perceptual standard for surface gloss. Here, we propose an experimental framework for making this problem tractable, starting from the premise that any perceptual standard of gloss discrimination must account for how distal scene variables influence the statistics of proximal image data. With this goal in mind, we rendered a large set of images in which shape, illumination, viewpoint, and surface roughness were varied. For each combination of viewing conditions, a fixed difference in surface roughness was used to create a pair of images showing the same object (from the same viewpoint and under the same lighting) with high and low gloss. Human participants (*N* = 150) completed a paired comparisons task in which they were required to select image pairs with the largest apparent gloss difference. Importantly, rankings of the scenes derived from these judgments represent differences in perceived gloss independent of physical reflectance. We find that these rankings are remarkably consistent across participants, and are well-predicted by a straightforward Visual Differences Predictor (Daly, 1992; Mantiuk, Hammou, & Hanji, 2023). This allows us to estimate bounds on visual discriminability for a given surface across a wide range of viewing conditions.

## Introduction

Determining visual thresholds for proximal stimulus variables—such as luminance (Nachmias & Kocher, 1970), wavelength (Pokorny & Smith, 1970), contrast (Campbell & Robson, 1968), orientation (Appelle, 1972), or spatial frequency (Campbell, Nachmias, & Jukes, 1970)—is conceptually straightforward, with well-defined psychophysical methods underpinned by signal detection theory (Green & Swets, 1966). Yet, it also often happens that we want to know how well participants can distinguish between stimuli that differ in some distal physical property, such as surface gloss. For example, in the pigment and paint industry, it is often necessary to manufacture parts with matching surface appearance, which would require differences in appearance to be ‘within tolerance,’ that is, below threshold (for a recent review and commentary, see European Coatings Dossier on Testing and Measuring, 2019)*.* Both research and development and quality control require some means to establish whether two samples are perceptually indistinguishable in terms of their gloss. Ideally, it should be possible to do this on the basis of a physical measurement applied to the surfaces. Similarly, computer graphics researchers often need to know how sensitive participants are to reflectance parameters, to determine, for example, whether a given approximation is acceptable (Greenberg et al., 1997; Pellacini, Ferwerda, & Greenberg, 2000). And in vision research, establishing discrimination thresholds for reflectance properties would also be useful for characterizing human perceptual abilities and constraining theories of gloss perception.

**Figure 1.**
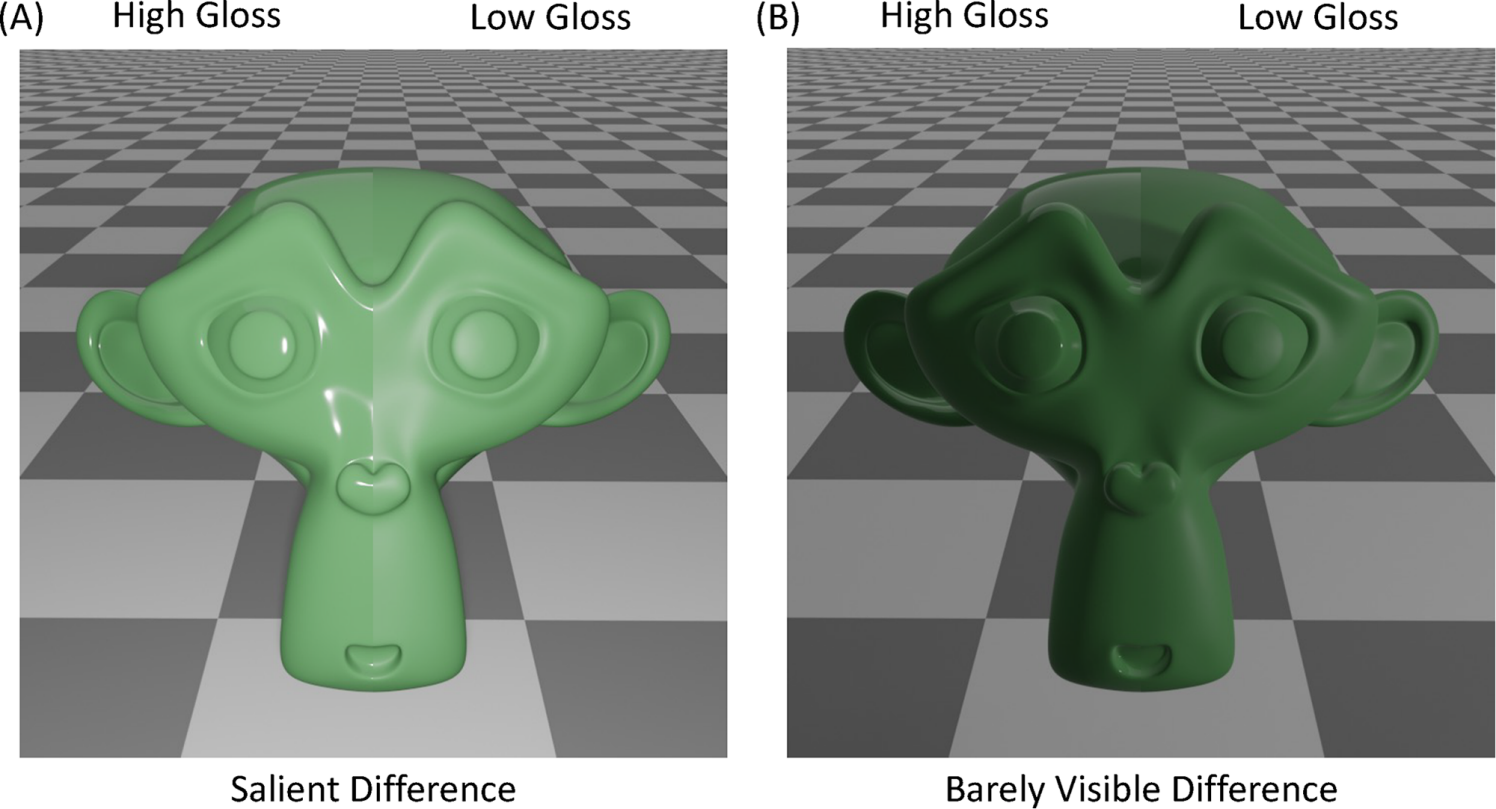
Identical differences in surface reflectance can be salient (**A**) or barely visible (**B**) depending on lighting direction. To create this effect, a point light source was shifted from the center to the side of the object.

Although the idea of measuring discrimination thresholds for gloss seems intuitive enough, there is a fundamental challenge, due to the fact that surface reflectance is a distal scene property, rather than a proximal stimulus variable like luminance or cone excitation ratios. The images that form the basis of any threshold measurements are the result of complex interactions between multiple distal scene factors in addition to the reflectance: the illumination striking the surface, the surface's shape and the observer's viewpoint. It is not possible to ‘leave out’ any of these factors; designating values for each factor is a prerequisite for creating the images required for the experiment. Nonetheless, lighting, shape and viewpoint can have potentially enormous effects on the measured thresholds (e.g., Zhang, de Ridder, Barla, & Pont, 2019, Zhang, de Ridder, Barla, & Pont, 2020). Under one set of conditions, a given difference in surface reflectance can significantly alter many pixels in the image, yielding very low threshold estimates (Figure 1A). Yet under other view conditions, the exact same difference in reflectance could have little to no effect on the image and therefore preclude the estimation of thresholds (Figure 1B). Thus, although we can experimentally determine whether any two images of surfaces are perceptually distinguishable, we do not know how the results generalize to other conditions. In concrete terms: Gloss thresholds measured under one illumination may be useless for determining whether two surfaces are perceptually distinguishable under a different illumination. The same holds for changes in shape or even viewpoint. Here, we seek to provide an approach to circumvent this challenge to yield ‘reasonable bounds’ on discrimination thresholds for gloss.

The practical needs of industry have driven the development of numerous color spaces over the past century. One of the most well-known, the CIE 1931 XYZ color space, was computed from simple color-matching experiments, where participants adjusted the relative intensities of three primary colored lights to produce an additive mixture that matched the color appearance of a monochromatic test light (Smith & Guild, 1931). Later in 1942, David MacAdam published the results of similar experiments that showed how sensitivity to differences in chromaticity vary within the 1931 CIE XYZ color space. Errors in color-matching performance were found to vary systematically within the space, thus indicating that equal increments within the space do not correspond to equal differences in perceived color. This motivated later researchers to propose color spaces that partially correct for such distortions (e.g., CIELAB). A perceptually-uniform space for gloss would be especially useful for industrial applications, where there is a need to maintain a consistent material appearance throughout the manufacturing process. However, such a space has remained elusive, owing to the multidimensional nature of gloss, and a lack of agreement about which dimensions of gloss are relevant for particular applications. The six attributes of perceived gloss set out by Hunter and Harold (1987) have been highly influential, and other results suggest that two to four dimensions account for nearly all variance in subjective comparisons of gloss, at least for the range of surfaces that were considered (Kildau, 2016; Pellacini et al., 2000; Prokott, 2016; Toscani, Guarnera, Guarnera, Hardeberg, & Gegenfurtner, 2020). However, the number and nature of these dimensions will depend on the surface appearances and viewing conditions chosen for testing, and the intended application. For example, human observers can accurately infer lighting properties and integrate them into their expectations of object appearance (Koenderink, Pont, van Doorn, Kappers, & Todd, 2007; Xia, Pont, & Heynderick, 2017), yet perceived surface reflectance can vary significantly with changes in higher-order illumination statistics (Motoyoshi & Matoba, 2012). Although low-order components of light fields remain relatively stable across scenes and correlate with scene geometry (Mury, Pont, & Koenderink, 2007, Mury, Pont, & Koenderink, 2009), observers can also adjust these components to match objects to the prevailing illumination so that they appear to be naturally integrated into their environment (Xia et al., 2017). This can in turn influence the perception of material qualities such as surface texture and reflectance. Additionally, as shown by Zhang et al. 2019, Zhang et al. 2020, higher-order components of light fields can be used to characterize how different lighting environments influence gloss perception. Although much attention has been given to understanding biases in gloss perception, in which lighting, shape, surface color, or surrounding context influences the overall level of gloss (e.g., Fleming, Dror, & Adelson, 2003; Hansmann-Roth, Pont, & Mamassian, 2017; Hansmann-Roth & Mamassian, 2017; Motoyoshi & Matoba, 2012; Nishida & Shinya, 1998; te Pas & Pont, 2005; Vangorp, Laurijssen, & Dutré, 2007), here we seek to define conditions for measuring sensitivity to changes in surface reflectance, paving the way for standards that could serve both industry and vision researchers.

Previous researchers have attempted to characterize gloss perception using a variety of experimental and analytical frameworks, traditionally with real surfaces in controlled lighting environments. For example, Obein, Knoblauch, and Viéot (2004) assessed the relationship between the perceived gloss of real surfaces and instrumental measurements of specular reflection using Maximum Likelihood Difference Scaling (Maloney & Yang, 2003). Although they did not find statistical evidence that a single scale could be used for both of the incident angles tested, one of their central claims is that participants’ judgments of gloss exhibit constancy under changes in viewing angle. However, the evidence for gloss constancy is rather mixed (Chadwick & Kentridge, 2015; Doerschner, Boyaci, & Maloney, 2010; Faul, 2019; Fleming et al., 2003; Olkkonen & Brainard, 2011), and it is not obvious how instrumental measurements can possibly generalize much beyond the original scene configuration, especially when shape and illumination are varied in addition to changes in viewpoint. It has long been known within the field that measuring the proportion of reflected light at a sparse sampling of incident angles is an unreliable predictor of perceived gloss (Harrison, 1945). Nevertheless, despite well-documented shortcomings, gloss meters based on this principle remain the industry standard, in part because such measurements can be collected quickly and better methods are not widely available. On the other extreme, one can measure reflected light at many more incident angles, covering the entire hemisphere above the surface plane, and use this data to estimate a bidirectional reflectance distribution function (BRDF) (Nicodemus et al., 1977). Until very recently, measuring BRDFs has been too costly and inefficient for widespread practical application (Filip & Kolafová, 2019). Despite these recent technical advances, however, it is unlikely that our perceptions of gloss are based on a BRDF-like representation of surface reflectance. Indeed, we have argued that the brain generates heuristic representations, or ‘statistical appearance models’ of gloss appearance over a range of typical viewing conditions (Fleming, 2014; Fleming & Storrs, 2019).

Advances in computer graphics simulation over the previous three decades have allowed vision researchers to apply these technologies to the study of gloss perception. For example, the study by Pellacini et al. (2000) is notable for its application of multidimensional scaling (Borg & Groenen, 2005) to judgments of glossy spheres shown in simulated illumination. With this data, they constructed a perceptually-uniform gloss space consisting of two dimensions (contrast and distinctness of the reflected image), which they later used to derive just-noticeable differences (JNDs) in gloss (Ferwerda, Pellacini, & Greenberg, 2001). Although these authors were the first to apply this approach to understand gloss perception, the generalizability of their results is limited to the set of appearances used to create the space (Fores, Fairchild, & Tastl, 2014). Given that shape and illumination strongly influence material appearance (e.g., Vangorp et al., 2007; Zhang et al., 2019, Zhang et al., 2020), what is a sufficiently diverse set of conditions for the purpose of characterizing gloss sensitivity? In the limit, iteratively rendering many combinations of illumination, shape, viewpoint, and surface reflectance will yield a set of images that includes the typical appearance of glossy surfaces across multiple material categories. However, in an industrial manufacturing context (e.g., quality control for surface coatings), often the goal is to measure appearance changes between multiple copies of a single material formulation. Our previous study (Cheeseman, Ferwerda, Maile, & Fleming, 2021) investigated gloss perception in such ‘symmetric’ viewing conditions, where we measured sensitivity to differences along a single perceptually-uniform dimension (specular reflectance), showing that, even with all other variables held constant, sensitivity varies significantly with stimulus magnitude. Here, we pursue a complimentary approach—holding surface reflectance constant while varying illumination, shape, and viewpoint—to identify viewing conditions that provide the most consistent and broadly applicable estimates of sensitivity. The current study therefore seeks to establish a framework for characterizing sensitivity to gloss per se, and perhaps, to other qualities of material appearance. To anticipate, we show that under symmetric conditions—when all scene parameters except reflectance are held constant—gloss discrimination reduces to an image discrimination task that can be well predicted by extant image-discrimination models. As a result, we can predict the variations in gloss discrimination that occur as various scene parameters are altered. This provides a route into defining ‘reasonable bounds’ bounds on gloss discrimination across viewing conditions.

## Experiment 1: Predicting apparent gloss differences across viewing conditions

Many studies have assessed gloss perception by varying the reflectance of surfaces under different viewing conditions. Here, we present participants with a fixed difference in surface reflectance while varying the illumination, shape, and viewpoint, with the intent of identifying an image metric that can predict perceived differences of gloss across viewing conditions. Importantly, because the difference in reflectance is identical across conditions, any visible differences in gloss are due to extrinsic distal variables that are independent of intrinsic surface reflectance. If an image metric can predict which viewing conditions tend to accentuate or obscure apparent gloss, this could provide a principled basis for establishing tolerances on gloss sensitivity in real world conditions.

### Methods

#### Participants

One hundred fifty adults (79 males and 71 females; age range, 18–68 years; *M* = 27 years, *SD* = 8 years) with normal or corrected-to-normal visual acuity participated in the experiment and were paid €10 per hour. Participants were recruited online using Prolific (prolific.co); they were required to have native fluency in English and a desktop or laptop computer. All experimental procedures were approved by the Justus Liebig University Giessen Psychology Department Ethics Board and conformed with the guidelines of the American Psychological Association (Version 2017) and the Declaration of Helsinki (Version 2013, excluding pre-registration). Informed consent was obtained from all participants.

#### Stimuli

Stimulus images were created with the Mitsuba physically-based renderer (Jakob, 2010). High dynamic range, linear RGB renderings were tone-mapped to low dynamic range sRGB images using the method described in Reinhard, Stark, Shirley, and Ferwerda (2002). The same tone-mapping operator was used for all images. Parameters controlling the overall luminance (key) and clipping of highlights (burn) in the image were set to the Mitsuba-default values of 0.18 and 0, respectively. Each 720 × 720 pixel image subtended approximately 7.7° of visual angle at a viewing distance of 50 cm, although viewing distance could not be controlled in the online experiment. Instead, participants were instructed to place a credit card (or another card of equivalent size) on their display screen, and adjust the length and width of a rectangle to match the size of the card. This measurement was used to calibrate the size of the images such that they were approximately the same size for different displays (see https://pavlovia.org/Wake/screenscale).

**Figure 2.**
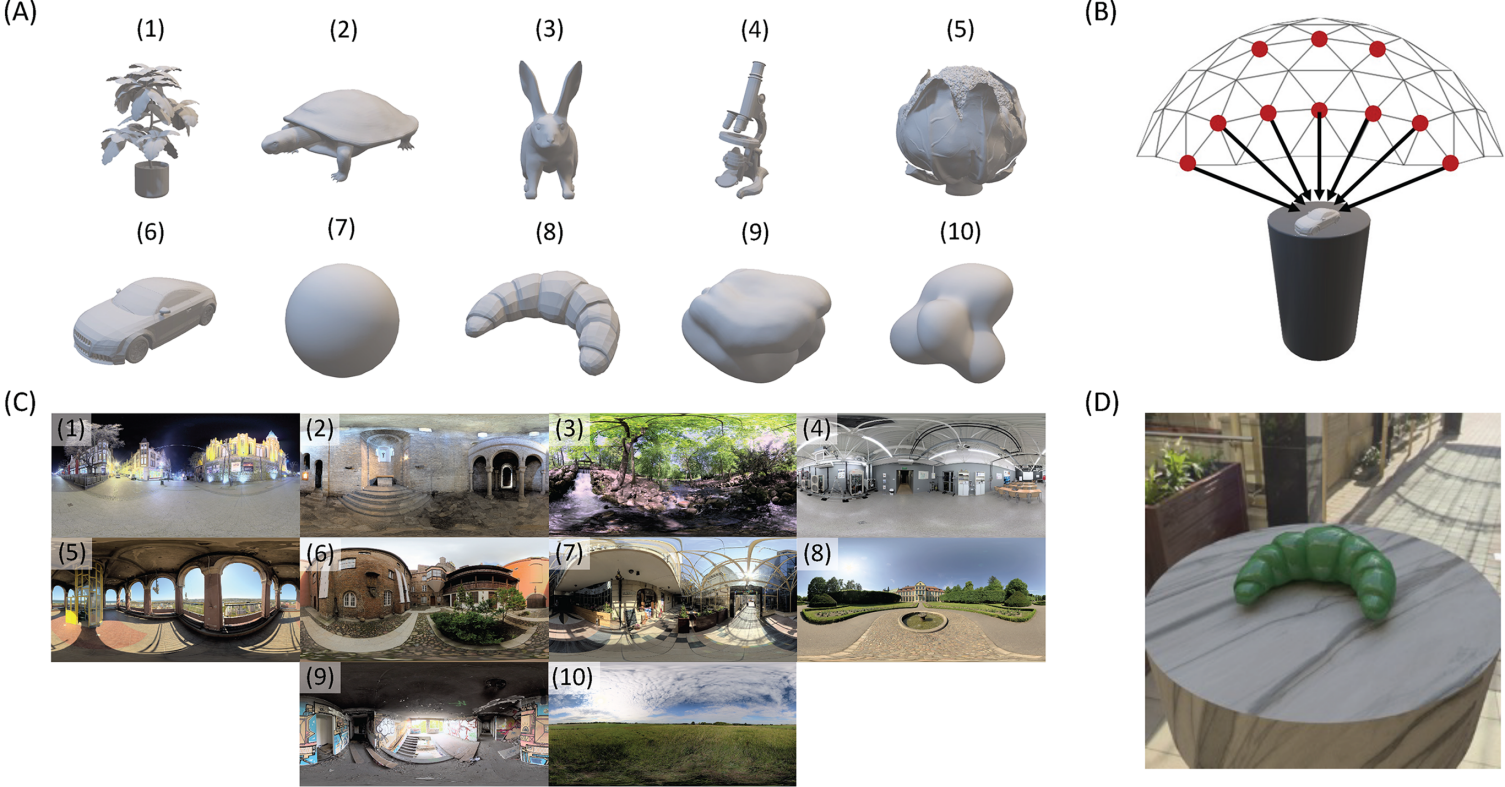
Scene variables that were used to create the stimulus images, including shapes (**A**), viewpoints (**B**), and illumination conditions (**C**). An example scene is shown in (**D**).

The basic scene (e.g., see Figure 2D) includes a central target object seated on a marble-textured pedestal under natural illumination. A set of 10 target objects (Figure 2A) was selected that span a variety of surface features that are more or less likely to accentuate gloss appearance. For example, some objects featured smoothly curved surfaces (e.g., car or turtle), whereas others featured rough or discontinuous surfaces (e.g., cabbage or plant). All objects in the scene were rendered with an improved version of the Ward BRDF model that obeys energy conservation and has better physical accuracy at grazing angles (Geisler-Moroder & Dür, 2010). The model has three parameters that control the specular reflectance (ρ*_s_*), diffuse reflectance (ρ*_d_*), and roughness (α) of a surface. The apparent glossiness of the target object was varied with two levels of surface roughness (α = 0.01; 0.19), and the specular and diffuse reflectance were fixed (ρ*_s_* = 0.066; ρ*_d_* = 0.1, 0.3, 0.1). A set of 10 high dynamic range environment maps (Figure 2C) was selected that featured a variety of indoor and outdoor illumination conditions. For example, environments with direct lighting can lead to bright, distinct highlights on a reflecting surface, whereas environments with diffuse lighting usually do not. Similarly, 10 evenly distributed viewpoints (Figure 2B) were sampled from the vertices of a hemi-icosphere positioned above the target object, thus providing a variety of high and low viewing angles for each target object. The combination of 10 shapes, 10 illuminations, and 10 viewpoints produced a set of 1,000 scenes. A subset of 100 scenes was randomly sampled (without replacement) from this larger set for use in Experiment 1.

#### Procedure

The experiment was created in PsychoPy v2021.2.3 (Peirce & Macaskill, 2018) and run on the Pavlovia experiment hosting platform (pavlovia.org). To avoid requiring participants to pre-load a large set of images at the start of the experiment, 100 scenes were further divided into 10 subsets of 10 scenes. Separate groups of 15 participants were recruited to judge each subset of scenes; these can also be understood as 10 independent experiments with separate groups of participants and stimuli. The first stage of the experiment required participants to complete 20 practice trials in a simplified version of the task using luminance patches rather than rendered images. For each practice trial, participants were instructed to select the left or right pair of images (arranged in a 2 × 2 grid on screen) that showed the larger difference in luminance, inspired by the method of quadruplets from Maximum Likelihood Difference Scaling. Seven participants failed to correctly judge these exaggerated suprathreshold differences in luminance with at least 90% accuracy during the practice phase and were excluded from the analysis, because it was assumed that they either did not understand the task instructions or a technical problem impeded their performance.

The task remained the same during the experimental trials, except that participants selected the left or right pair of images (rather than luminance patches) in which there is a larger difference in apparent gloss of the target object. That is, observers compared relative gloss differences within fixed scene configurations, minimizing possible contextual effects (e.g., see Hansmann-Roth et al., 2017; Hansmann-Roth & Mamassian, 2017) by ensuring that gloss variations were evaluated against the same background. Additionally, to account for outliers, participants who completed the experimental trials were excluded if their responses produced an extremely low correlation with other participants’ judgments of the same set of images. Frequencies representing how often each scene is chosen were calculated for each participant and compared across participants. If the average correlation between one participant's frequencies and those of the other participants exceeds the interquartile range of these average correlations (multiplied by 1.5), this was considered an outlier, and the participant's data were excluded from the analysis. Twenty-two outliers were excluded in total. In summary, separate groups of 15 participants judged separate sets of 10 scenes. For each set of scenes, 45 unique scene pairs were presented in a random order across 6 repetitions. One hundred fifty participants collectively completed a total of 40,500 trials. All data and stimuli have been made available in a public repository maintained by the Open Science Framework (https://doi.org/10.17605/OSF.IO/2HYN5).

#### Image metrics

HDR-VDP-3 (Mantiuk, Hammou, & Hanji, 2023) is a popular metric for predicting the visibility of image differences and assessing the impacts of compression or other image processing operations on image quality. In our analysis, HDR-VDP-3 was used to predict perceived differences between test and reference images (i.e., of the same surface with high vs. low roughness). The metric was applied in a side-by-side task mode, which is appropriate for comparing two images displayed adjacent to each other. Input images were encoded in sRGB-display format to correspond with standard color images displayed on an sRGB monitor, with peak luminance calibrated to 100 cd/m^2^ and a black level at 1 cd/m^2^. The images were processed with a high angular resolution of 120 pixels per visual degree, appropriate for a close viewing distance or high-resolution display. For the modulation transfer function, which models the scattering of light in the eye's optics—referred to as glare—we chose to bypass this step by setting the 'mtf' option to 'none.' This decision was made because the glare effect, although significant for high-contrast HDR images, adds computational complexity that was not essential for our purposes, given that the input images were low-dynamic range. The output of HDR-VDP-3 provided us with a probability map of detection for each pixel (*P_map_*), with values ranging from 0 to 1. We computed the mean of this probability map to represent the visibility metric for each stimulus condition, allowing us to assess the average detectability of image differences across the entire image, including the target object and background, as in many contexts (e.g., photographs) no object mask is available. Although HDR-VDP-3 also provides a single valued probability of detection (*P_det_*) for the whole image (by taking the maximum value of *P_map_*; see Mantiuk, Kim, Rempel, & Heidrich, 2011, p. 6), we found that the average of the probability map (*P_map_*) was a better predictor of the human data.

Unlike HDR-VDP-3, which can predict visible differences from full sRGB images, our measurements of contrast, coverage, sharpness, and skewness (similar to previous studies; see Marlow et al., 2012; Motoyoshi et al., 2007) were derived by first converting the sRGB images into luminance images (calibrated to cd/m^2^). We then eliminated the diffuse component, thus ensuring the metrics were computed only from specular reflections. Subsequently, to remove reflections from within the object or from the pedestal, we thresholded the specular image. Pixels exceeding a certain intensity threshold—determined as a percentage (*k%*) of the highest intensity, with *k* values set at 0, 1, 3, 5, 10, 20, 30, and 40—were retained. The *k* values were selected to evaluate a range of intensity thresholds.

Following thresholding, we then decomposed the thresholded highlight image into eight sub-band images through Gaussian band-pass filtering across a range of frequencies. This allowed us to capture the effects of spatial frequency modulation on the perception of gloss (Boyadzhiev et al., 2015). The contrast for each frequency band, as well as for the combined frequency image, was determined by calculating the root-mean-square-error (*RMSE*) of the pixel intensities. Alongside contrast, we also evaluated metrics for highlight coverage and sharpness, which were calculated from the thresholded highlight images. Coverage is the proportion of the object area that is covered by specular reflections, providing an indication of the extent of gloss across a surface. Sharpness is defined by the rate of change in luminance, measured using the slope of the local magnitude spectrum and local maximum total variation (TV) to emphasize areas of the object where intensity transitions are most pronounced (Vu et al., 2012). The values of k (for contrast, coverage, and sharpness) and spatial frequency bands (for contrast only) were varied to determine values that produced the best correlation with the human data. All of these image metrics were calculated with the scene background masked, leaving only the object region.

Whereas Marlow, Kim, and Anderson (2012) obtained estimates of contrast, coverage, and sharpness through empirical observer judgments, our approach—like that of Schmid, Barla, and Doerschner (2023) and Morimoto et al. (2023)—relies on objective, image-based metrics. It is important to note that this approach to segmenting specular reflections by luminance-based thresholding may exclude non-highlight (i.e., indirect) specular reflections that fall below the threshold, and thus potentially underestimate specular coverage on highly glossy surfaces. However, despite this limitation, such metrics have been shown to correlate robustly with perceived gloss across a variety of shapes, materials, and lighting environments (Morimoto et al., 2023; Schmid et al., 2023).

### Results

**Figure 3.**
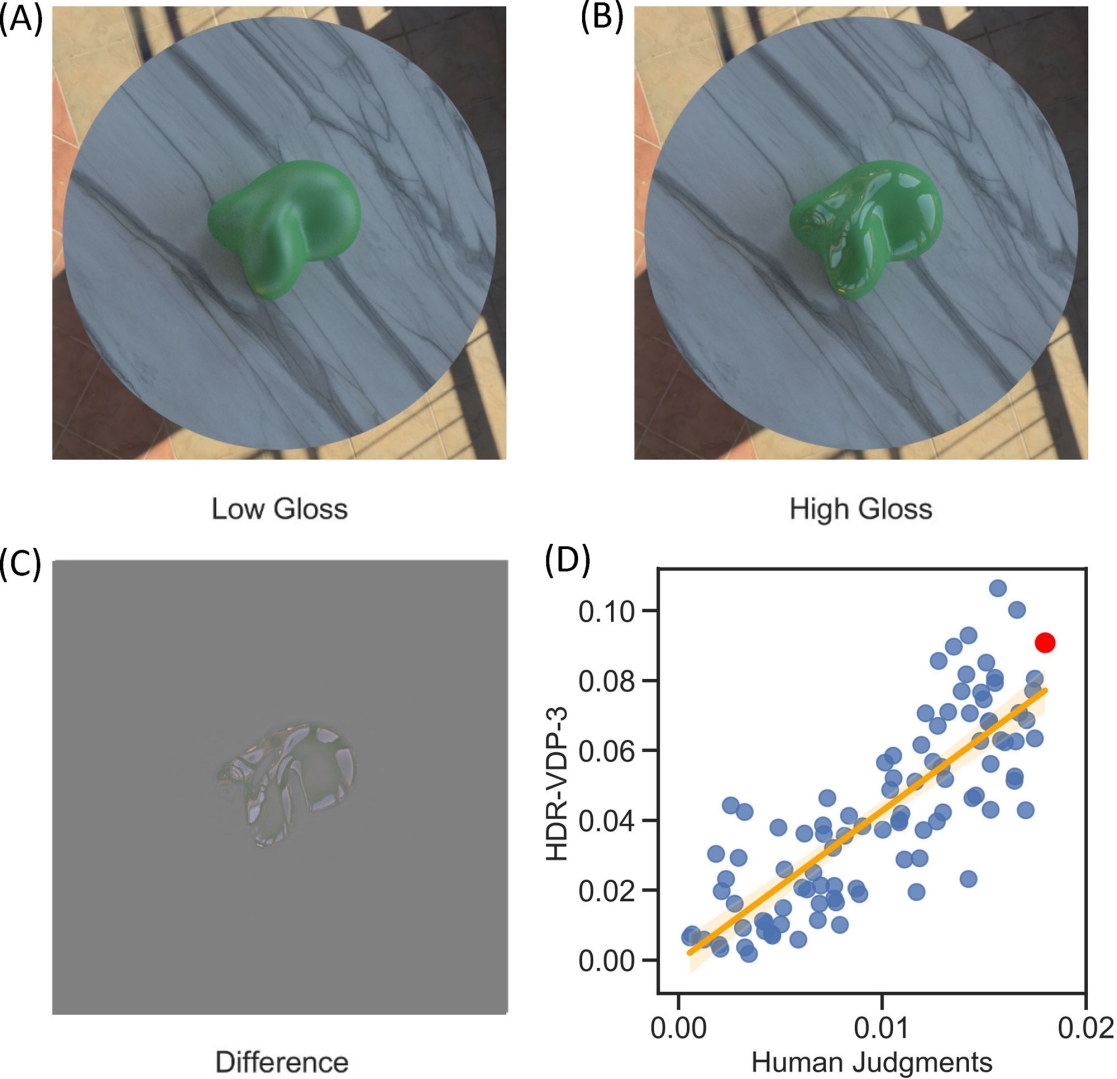
The scene with the largest visible difference (**A** and **B**), together with the luminance difference between the high- and low-gloss images (**C**). The scatterplot (**D**) shows a correlation of 0.81 between the HDR-VDP-3 predictions (arbitrary units) and human judgments (proportion of trials each scene was chosen). Each datapoint in the scatter plot represents one of the 100 scenes; the datapoint highlighted in red corresponds to the scene with the largest visible difference (**A** and **B**).

The scene that most participants judged to depict the largest visible difference in apparent gloss is shown in Figure 3, along with *RMSE* difference across RGB channels between the two images, and predictions of one image metric (High Dynamic Range Visual Difference Predictor; Mantiuk et al., 2023) versus human judgements for all stimuli. Apparent differences in gloss caused by variations in lighting, shape, and viewpoint were well predicted by this image metric, which produced a correlation of 0.81 with the behavioral data (i.e., how often each scene was selected for showing a larger gloss difference). An even better predictor of the human data (*r* = 0.87) uses the mean of the *P_map_* limited to the object region of the image (see Object Size Analysis in the Supplementary Information). Impressively, this object-only version of the metric explains an additional 10 percentage points of variance (*R^2^* from 0.66 to 0.76 for the full-image and object-only metric, respectively). Yet, such object masks are not available in many real-world conditions, so for further analysis and experimentation, we focused on the full-image version of the model, which therefore provides a conservative estimate of how well we can predict discrimination.

**Figure 4.**
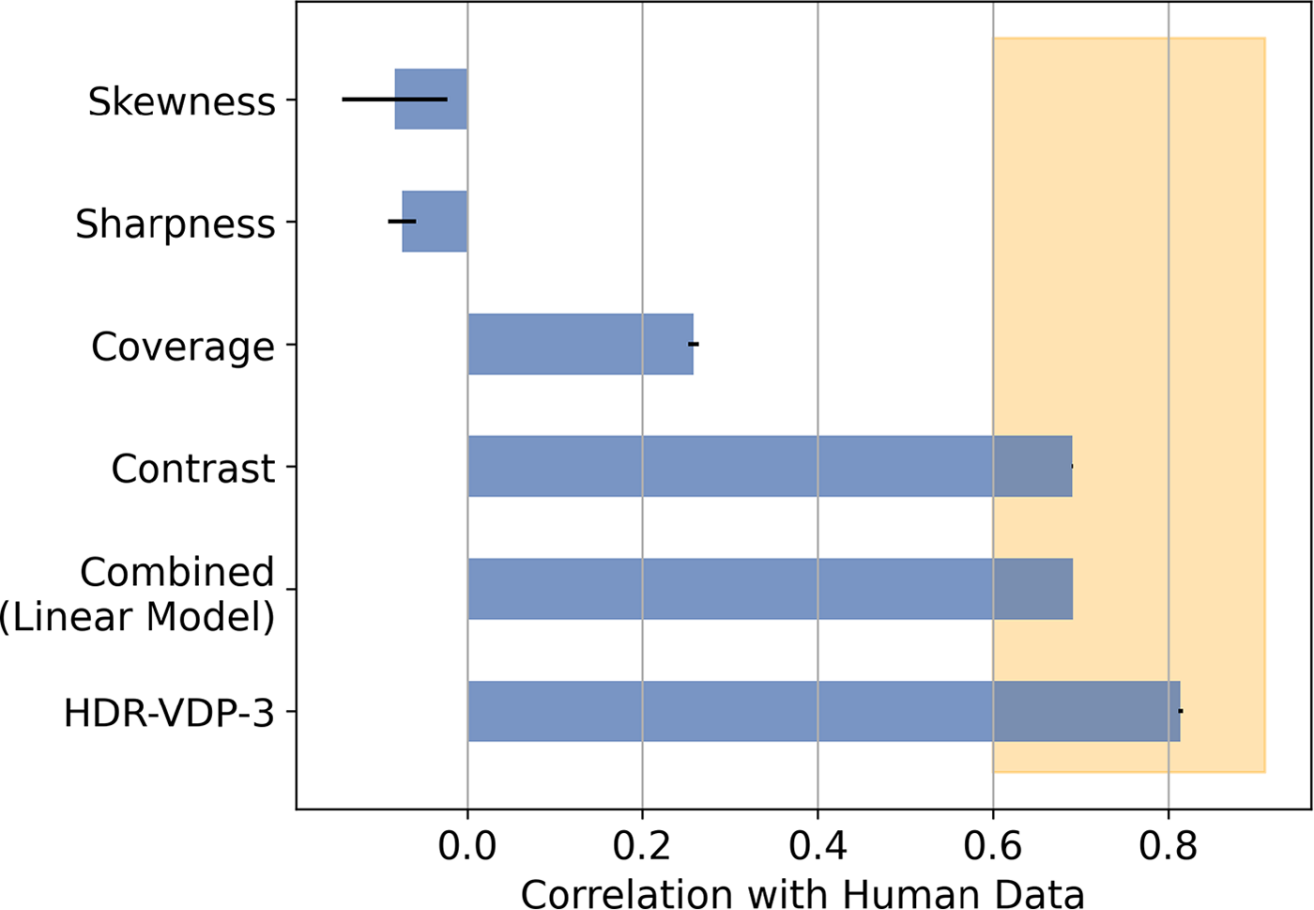
Correlations between human gloss judgments and individual image metrics, as well as a linear model of sharpness, coverage, and contrast. Error bars represent the standard error of the mean. The interparticipant correlations span the range highlighted in orange, with an average of 0.83.

Other image metrics were also evaluated for their ability to predict these data, including sub-band contrast, highlight coverage, highlight sharpness, and the skewness of the pixel intensity histogram. These metrics have all been shown to correlate with gloss appearance in experimental conditions (Anderson & Kim, 2009; Kim & Anderson, 2010; Morimoto et al., 2023; Motoyoshi, Nishida, Sharan, & Adelson, 2007; Schmid et al., 2023). A generalized linear model was used to assess the relationship between these metrics and human judgments. The model included HDR-VDP-3, contrast, coverage, sharpness, and skewness as predictor variables. The analysis revealed that only HDR-VDP-3 (coefficient = 0.1215; standard error = 0.013; *z* = 9.603; *p* < 0.0001) and contrast (coefficient = 0.2645; standard error = 0.054; *z* = 4.942; *p* < 0.0001) were significantly correlated with human judgments (Figure 4).

Additionally, to assess whether a linear combination of image cues could better predict perceived gloss, we fit a multiple linear regression model using contrast, coverage, and sharpness as predictors of the human gloss ratings. All predictors were z-score normalized prior to model fitting to place them on a common scale. This approach follows the cue–combination logic used in prior studies (e.g., Qi, Chantler, Siebert, & Dong, 2014; Marlow & Anderson, 2013). The resulting model achieved a Pearson correlation of *r* = 0.69 (same as contrast alone; see Figure 4), indicating no substantial improvement in predictive power from combining cues. Inspection of the model weights confirmed that contrast dominated the prediction, while coverage and sharpness received near-zero weights. These results suggest that for our stimuli, contrast alone captures nearly all the explainable variance in human gloss judgments.

Although HDR-VDP-3 is optimized for the prediction of psychophysical data and is not meant to be a biologically plausible model of the human visual system, HDR-VDP-3 does explicitly model the optical and retinal transformations that occur in the first stages of human visual processing, as well as subsequent parsing of spatial frequency and orientation information in primary visual cortex. These features are used to model contrast masking and (neural) contrast sensitivity, and collectively influence the metric's assessment of image quality and visibility of image differences. Despite the complexity of HDR-VDP-3, its overall predictive power in our study appears to depend on simpler, more fundamental image attributes such as contrast.

## Experiment 2: Image metric validation in a laboratory-based control experiment

We have a metric (HDR-VDP-3) that predicts human judgments of gloss differences across variations in lighting, shape, and viewpoint. However, these judgments were collected from participants over the Internet in uncontrolled viewing conditions with unknown display characteristics, viewing distance, and ambient illumination, which may have influenced our results (e.g., see Haghiri, Rubisch, Geirhos, Wichmann, & von Luxburg, 2019). The purpose of this second experiment was, therefore, to test whether the predictions of the model are also valid for controlled laboratory conditions using scenes selected from Experiment 1.

### Methods

#### Participants

Twenty-two adults (7 males and 15 females; age range, 20–39 years; *M* = 27 years, *SD* = 5 years) with normal or corrected-to-normal visual acuity participated in the experiment and were paid €12 per hour. Participants were recruited from the university student population. All experimental procedures were approved by the Justus Liebig University Giessen Psychology Department Ethics Board and conformed with the guidelines of the American Psychological Association (Version 2017) and the Declaration of Helsinki (Version 2013, excluding pre-registration). Informed consent was obtained from all participants.

#### Stimuli

**Figure 5.**
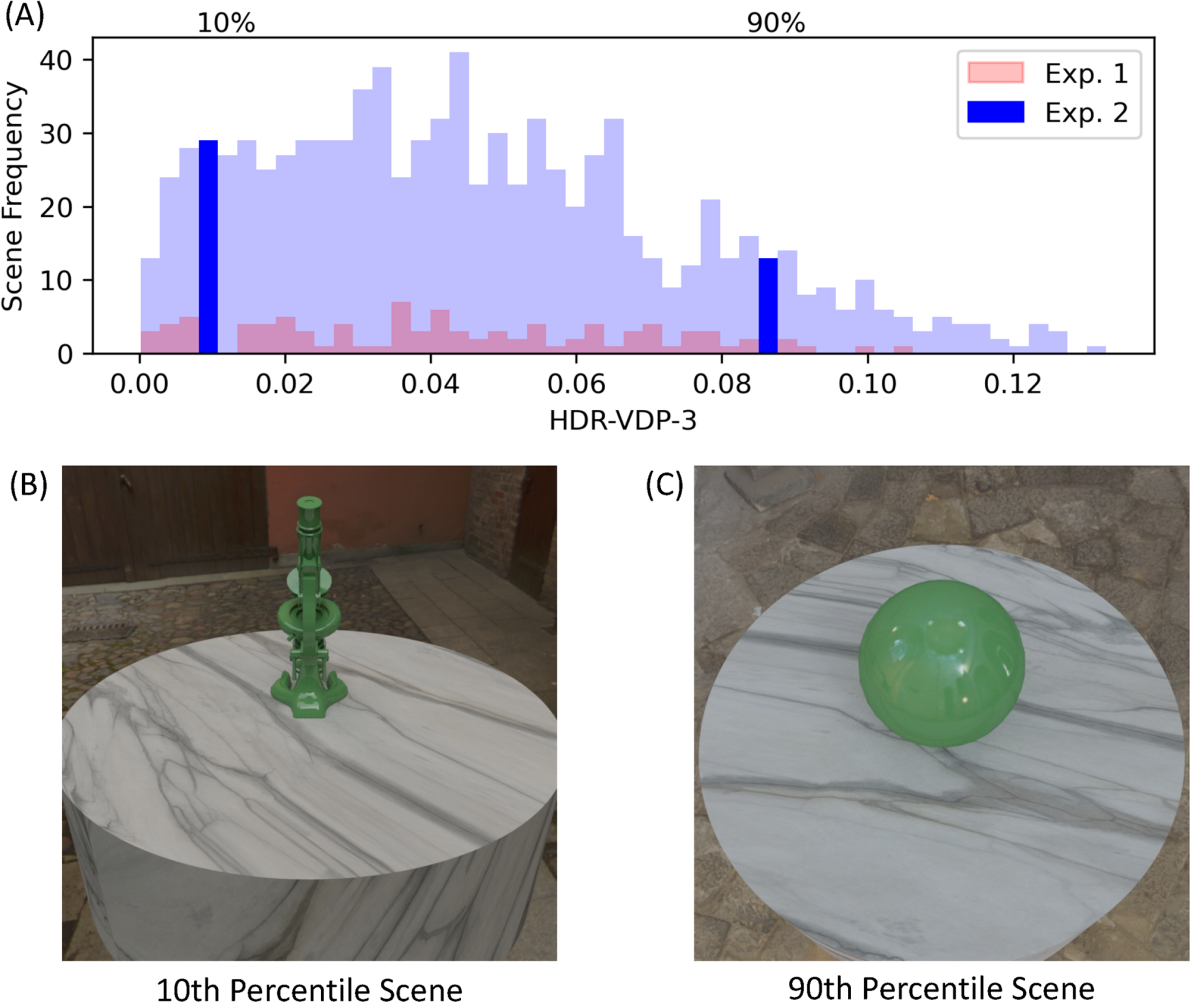
A histogram of the HDR-VDP-3 predictions for the full set of images (**A**). Images used in Experiment 1 are highlighted in red. Images used for Experiment 2 (**B** and **C**) were selected from the 10th and 90th percentile bins of the histogram, shown highlighted in dark blue.

**Figure 6.**
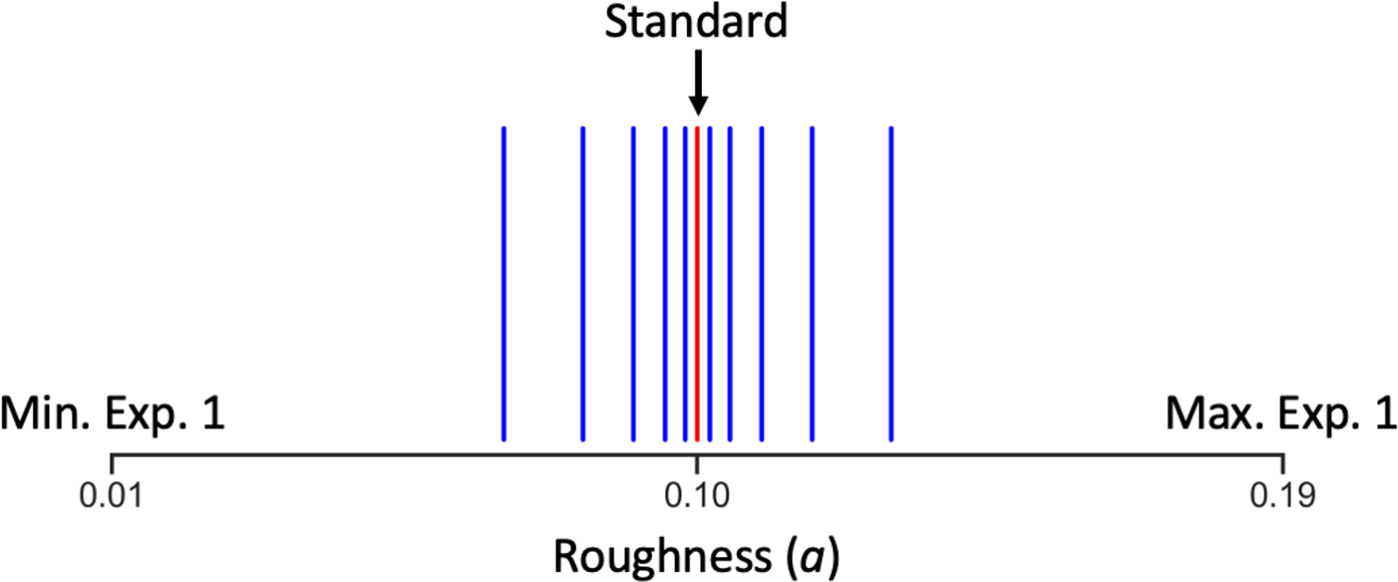
Roughness (α) parameter values of the Ward model used to create stimulus images for Experiment 2. The values of roughness used to create the low and high gloss images in Experiment 1 correspond with the minimum and maximum values of the range shown here.

The distribution of HDR-VDP-3 predictions for the full set of images is shown in Figure 5. As previously mentioned, we used a subset of 100 scenes for the first experiment (red bars). For the second experiment, we selected two new scenes from the full set of 1,000 scenes (dark blue bars). Specifically, using the version of the model that takes the whole image as input (i.e., without masking the background), we chose two scenes where gloss sensitivity is predicted to be low or high, corresponding to the 10th and 90th percentile values of the distribution, respectively. For each scene, we rendered new images with finer differences in surface roughness using the log-spaced values illustrated in Figure 6 to validate the performance of the model for finer differences in roughness. The standard roughness value was 0.1, and test images were rendered with the following values of roughness: 0.0702, 0.0824, 0.0901, 0.0950, 0.0981, 0.1019, 0.1050, 0.1099, 0.1176, and 0.1298. All other scene parameters remained identical to those used in Experiment 1.

#### Procedure

The experiment was created in PsychoPy v2021.2.3 (Peirce & Macaskill, 2018) and run on a Dell Precision T3500 desktop computer. The images were presented in a dark room on an Eizo ColorEdge CG277 LCD monitor, which has a 68.58 cm diagonal length and a resolution of 2,560 × 1440 pixels. Each image subtended approximately 19° of visual angle from a viewing distance of 50 cm. The display was calibrated to the sRGB color gamut with an 80 cd/m² D65 white point and a gamma setting of 2.2. During each trial, participants were presented with two images arranged side by side, and their task was to select the (left or right) image that showed a green target object with a higher degree of gloss. Other than this difference in the apparent gloss of the target object, all other scene variables were identical between the two images. This two-alternative forced-choice task replicates the procedure used in our previous work (Cheeseman et al., 2021). The first stage of the experiment required participants to complete 10 practice trials with a pair of images (selected from Experiment 1) showing a clearly visible gloss difference. Feedback was provided during the practice trials to indicate whether the object in the chosen image had a higher gloss (i.e., lower roughness). All participants were able to complete the practice trials without difficulty and were allowed to proceed to the next phase of the experiment. In this second phase, participants performed the same two-alternative forced-choice task with images from two scenes that had not been shown in Experiment 1. Image pairs from each scene were presented repeatedly (40 times) in random order, following the method of constant stimuli; that is, for each scene, a standard image was presented alongside 1 of 10 comparison images (see Stimuli section). On average, the experiment lasted approximately 40 minutes, with a rest period at the halfway point. Collectively, our 22 participants completed 17,600 trials of this task. All data and stimuli have been made available in a public repository maintained by the Open Science Framework (https://doi.org/10.17605/OSF.IO/2HYN5).

### Results

**Figure 7.**
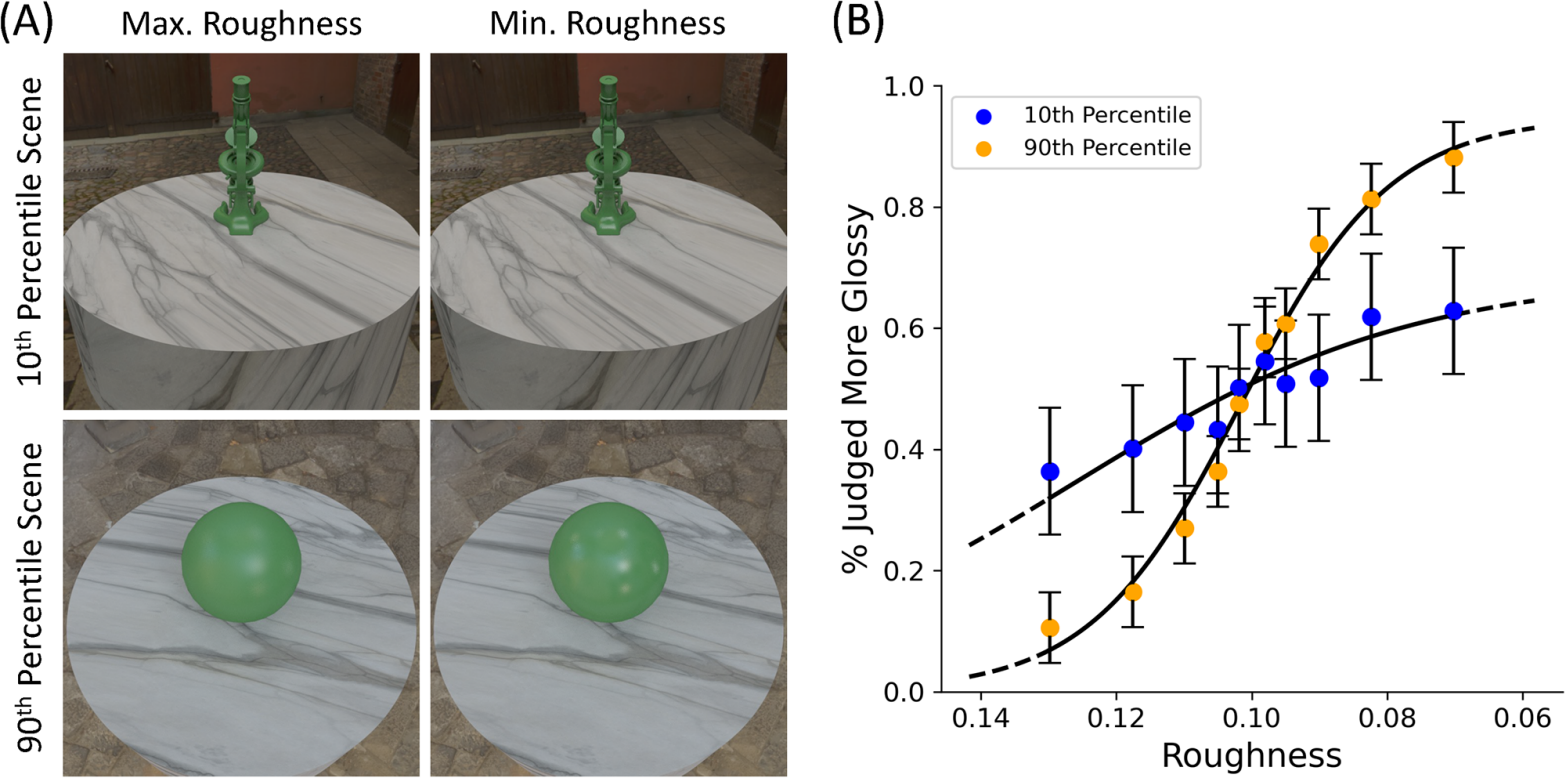
The minimum and maximum roughness images for the 10th and 90th percentile scenes (**A**), along with their corresponding psychometric functions calculated from pooled observer data (**B**). The significantly steeper slope of the psychometric function corresponding to the 90th percentile scene validates the prediction of HDR-VDP-3. Error bars signify the standard deviation of psychometric function fits across participants.

Figure 7 shows the minimum and maximum reflectance images for the 10th and 90th percentile scenes, along with their corresponding psychometric functions calculated from pooled observer data using *psignifit 4* (Schütt, Harmeling, Macke, & Wichmann, 2016). HDR-VDP-3 predicts lower sensitivity for the 10th percentile scene, and higher sensitivity for the 90th percentile scene, respectively. Note that, in Experiment 1, a fixed difference in roughness was predicted to be more visible with the combination of lighting, shape, and viewpoint shown in the 90th percentile scene. In the current experiment, smaller differences in roughness were used to measure discrimination performance in controlled laboratory conditions. The significantly steeper slope of the psychometric function for the 90th percentile scene (*M_slope_* = 12.65; *SE* = 6.46) compared with the 10th percentile scene (*M_slope_* = 14.89; *SE* = 11.84) validates the prediction of HDR-VDP-3—that participants would be more sensitive to the same reflectance difference when viewed with this combination of lighting, shape, and viewpoint.

#### Analysis of HDR-VDP-3 predictions

**Figure 8.**
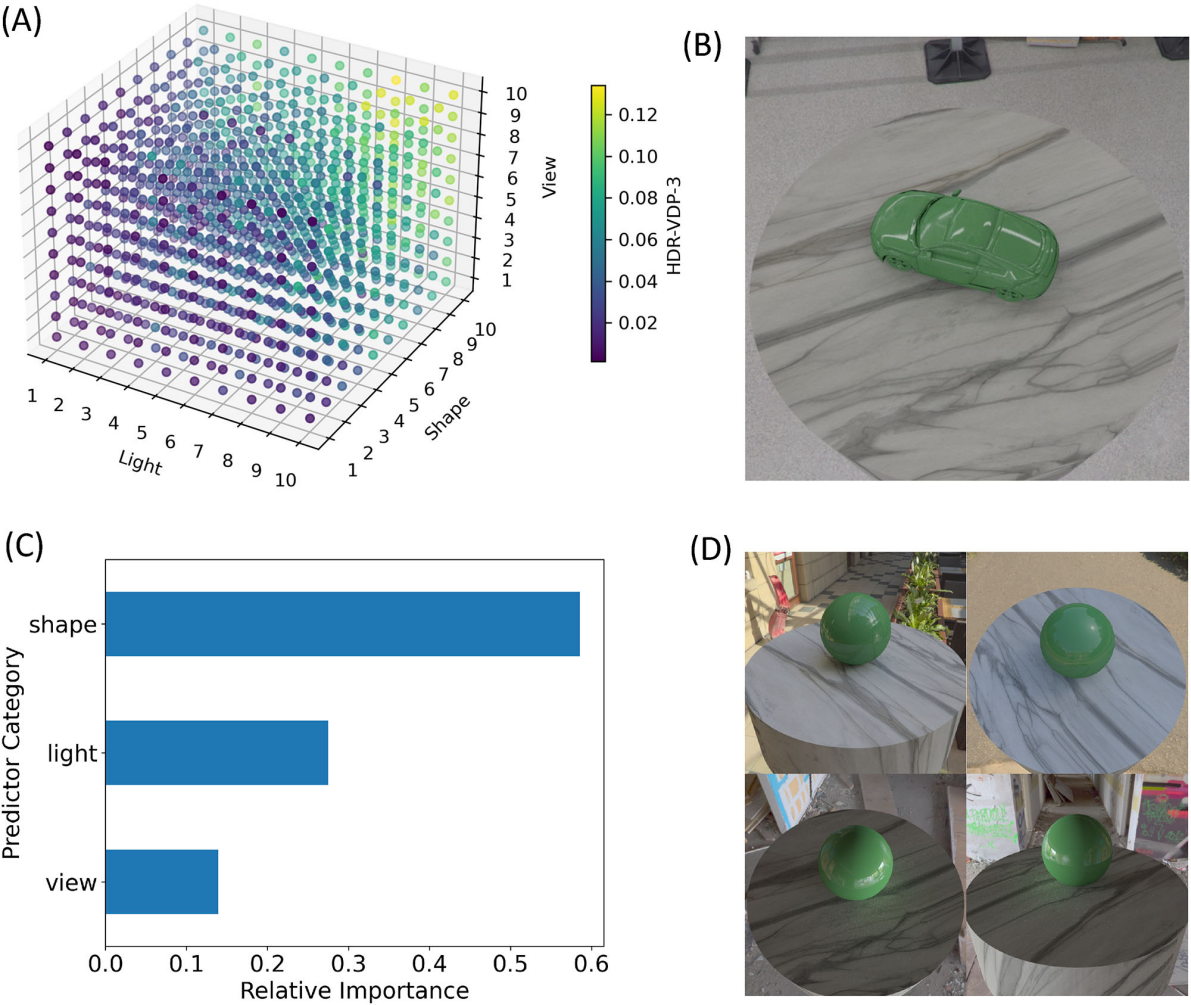
(**A**) Rank-ordered combinations of lighting, shape, and viewpoint that should yield the highest sensitivity to differences in reflectance, according to an analysis of our full image set using HDR-VDP-3. (**B**) The scene with the combination of these scene variables that yielded the highest predicted discriminability. (**C**) A random forest model was used to determine the overall importance score of each variable, revealing that, for our image set, object shape has the largest contribution to variance in discriminability. (**D**) According to this analysis, the sphere led to the highest variance in discriminability across different lighting environments and viewpoints.

**Figure 9.**
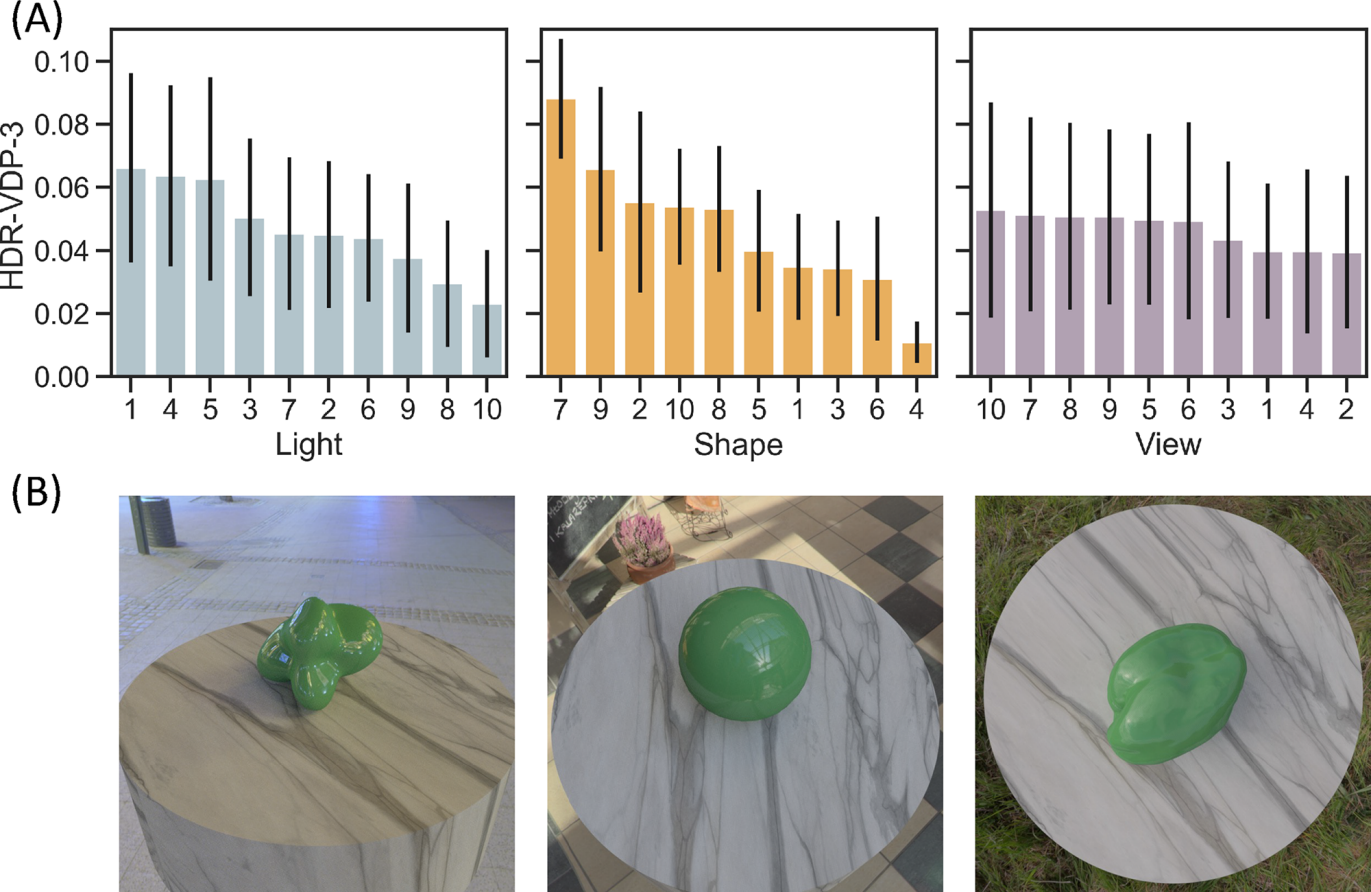
(**A**) Descriptive summaries of mean HDR-VDP-3 model predictions for individual lighting maps, object shapes, and viewpoints. Error bars represent standard deviation. (**B**) Example scenes that included the lighting, shape, or viewpoint that produced the highest mean value.

Given that HDR-VDP-3 has been validated in controlled laboratory conditions, the model predictions can be used to search the full image set for combinations of lighting, shape, and viewpoint that should yield the highest sensitivity, shown in Figure 8. To evaluate the relative effect of lighting, shape, and viewpoint on the model predictions, we used a random forest model (Breiman, 2001; Hastie, Tibshirani, & Friedman, 2009). Random forests are particularly suited for this analysis because they do not assume linear relationships and are better able to predict continuous values using categorical variables. In this context, the target variable (mean *P_map_* predicted by HDR-VDP-3) is estimated based on paths taken through a series of decision trees constructed from the categorical variables. The random forest algorithm considers every possible division of the HDR-VDP-3 prediction values for levels of each categorical variable, and calculates which path will result in the largest decrease in variance of these values. Feature importance scores from a random forest model indicate which categorical variables contribute most to estimating the target variable across all the decision trees. Figure 8C shows the overall importance of each variable, revealing that shape contributed the most to variability in model predictions. Furthermore, if we calculate descriptive statistics on the predictions of HDR-VDP-3, as shown in Figure 9, these can be used to estimate upper and lower bounds of predicted sensitivity for specific shapes (across lighting and viewpoint), or any other combination of these variables. A possible reason for the high importance of shape is that differences in proximal object size across shapes influenced detectability, since larger objects potentially provide more visual information. However, our analysis (see Object Size Analysis in the Supplementary Information) shows that object size accounts for relatively little variance in model scores.

## General discussion

In Experiment 1, we rendered a set of images with a fixed difference in surface reflectance (roughness) for a variety of different lighting conditions, object shapes, and viewpoints. We collected human judgments of these images in an online experiment, finding that participants were highly consistent in their ranking of gloss differences across these viewing conditions. An existing model that predicts the visibility of image differences, HDR-VDP-3, was able to predict the human ranking of the gloss differences in our image set to a surprising degree—in fact, well within the range of inter-participant correlations (Figure 4). This finding indicates that HDR-VDP-3 performed as well as any image-computable model could, given the variance in our data. Interestingly, in this case, similar performance can also be achieved by measuring the contrast of the specular term. In Experiment 2, HDR-VDP-3 was used to select two scenes from our full image set, representing combinations of lighting, shape, and viewpoint that represent opposing predictions of the model, leading to lower or higher sensitivity to the same difference in physical reflectance. The model predictions were validated in controlled laboratory conditions, evidenced by a significant difference in gloss sensitivity (see Figure 7). These model predictions were then used to estimate the relative contribution of specific viewing conditions to gloss sensitivity. This work provides a first step toward characterizing the impact of viewing factors on gloss discrimination, so that ‘reasonable bounds’ on JNDs can be established.

### Toward reasonable bounds on JNDs for surface reflectance

Our study suggests that HDR-VDP-3 can predict how gloss discriminability varies across a range of viewing conditions—at least, about as well as individual participants predict one another. This work lays the foundations for automatically establishing JNDs ‘within reasonable bounds’ for materials with particular appearances (e.g., coatings with particular formulations). Here we outline the approach and describe some of the additional open research questions that would need to be resolved to develop a working system.

The basic logic of the approach runs as follows. The JND for a given surface reflectance characteristic is defined as the smallest magnitude change in the physical BRDF of the surface that can be detected. As noted throughout this study, this value can vary due to extrinsic factors such as lighting, object shape, and viewpoint. Our goal is to predict the range of values the JND can take across ‘reasonable’ changes in viewing conditions.

As there are potentially an infinite number of possible changes to the BRDF, let us limit ourselves to the case where we wish to evaluate the JND for a specific kind of reflectance change. A simple case would be when varying a single parameter of an analytic BRDF model (as in our experiments), although any change that can be summarized with a single number suffices. For example, suppose a paint manufacturer wishes to determine tolerance bounds on a particular parameter of the paint formulation or manufacture process, such as the temperature at which a coating should be applied, or the duration of grinding of a particular ingredient in the paint. As long as the parameter leads to a smooth and systematic change in the BRDF—for example, by changing the specular lobe—in a predictable way, then we can use images of samples with different parameter values viewed under constant conditions to estimate a JND with HDR-VDP-3 (or some other image-computable image-difference metric).

To be more precise, the assumption is that (small) changes in the manufacture parameter shift the BRDF along a specific vector in the high-dimensional space of all BRDFs (e.g., making the specular lobe broader in a particular way). The goal of determining the tolerance for the parameter then becomes the goal of determining the magnitude of that vector for which two samples can just be discriminated.

In the unusual circumstances that the BRDF will be seen exclusively under fixed viewing conditions (i.e., a single, specific shape, under fixed specific lighting, from a specific viewpoint), then it should be sufficient to image samples (e.g., render or photograph) with a few values of the parameter under those viewing conditions, and run the resulting images through the image difference predictor. The JND will be inversely proportional to the change in the image difference metric caused by a given change in the reflectance. Although in this study we used only a single pair of values of reflectance properties to estimate the impact on the image metric, in practice, using multiple samples with different values would give more robust estimates of the impact of the reflectance parameter on the image differences and therefore a more reliable estimate of the JND.

However, more typically the extrinsic view parameters (shape, lighting, and viewpoint) are free to change. As extrinsic variables change, the impact of a given change in reflectance on the image also changes—making larger, more detectable image changes in some conditions, and smaller, less detectable ones in other conditions. As a result, there will be a distribution of values for the JND across extrinsic parameters. A route to estimating this distribution is to change the shape, lighting and viewpoint across a representative range of conditions, and for each one, image the surface with a range of reflectance parameters. Again, by passing the resulting images through HDR-VDP-3 (or other image-difference metric), it should be possible to predict the JND for each particular combination of extrinsic parameters.

Given a distribution of JND values, an empirically informed decision can then be made about ‘reasonable bounds’ for the JND. For example, one might select the 95th percentile of the distribution of values, meaning that two materials within the tolerance for that reflectance-determining parameter would look indistinguishable in at least 95% of conditions.

In general, the greater the strictness of the tolerance requirements, the more different lighting, shape, and viewpoint conditions would need to be evaluated to estimate the tail of the JND distribution. An alternative approach to sample the tail of the distribution more efficiently than random sampling would be to seek out ‘adversarial’ combinations of lighting, shape, and viewpoint that make the given differences in reflectance especially salient in the image (Bousseau, Chapoulie, Ramamoorthi, & Agrawala, 2011). A particularly efficient way of achieving this in computer graphics contexts would be to use differentiable rendering to optimize the predicted visible difference between surfaces by varying lighting, shape, and viewpoint. This would aid selecting tolerances based on ‘worst case’ scenarios. However, it is worth remembering that it is almost always possible to construct a particularly problematic combination of shape, lighting, and viewpoint, and such non-generic worst-case conditions may essentially never be encountered in the real world (Freeman, 1994). Depending on the derivatives of the scene parameters that lead to very small JNDs, the ‘worst case’ may require extremely precise alignment of the viewpoint with the surface and light sources, for example, which are unlikely to occur except under carefully contrived circumstances.

The proposed testing framework could be directly integrated into industrial workflows for applications, such as automated quality inspections and material standardization. One practical implementation could involve efficiently measuring an average BRDF from real surfaces and combining it with three-dimensional scans of manufactured objects to simulate their appearance under a standardized set of lighting environments (e.g., see Filip, Kolafová, & Vávra, 2022). Using a coarse sampling of different viewpoints, this approach would enable industries to systematically assess how surface properties, such as gloss, manifest across typical viewing conditions. For example, if two material samples—one high gloss and one low gloss—are captured and simulated, this could provide a means of estimating of how their perceived gloss difference would appear in real-world settings. Using image-based predictors, like HDR-VDP-3, could aid in automating the process of characterizing how discriminable the materials are. Such a system could enhance quality control processes by reducing reliance on gloss meters or subjective human assessments, ensuring consistency in product appearance across different lighting conditions and viewing angles.

### Limitations and future work

We have outlined a general approach to determining ‘reasonable bounds’ on JNDs for surface reflectance properties; however, there are many open research topics and additional steps to convert this outline into a working and validated system suitable for critical applications. Here we illustrated the ability of HDR-VDP-3 to predict the relative discriminability of a change in roughness (distinctness-of-image gloss) across changes in lighting, shape, and view angle. Future work should confirm that a similar approach is effective for other reflectance parameters. It would also be important to demonstrate that the approach generalizes beyond computer graphics to real-world conditions.

Previous studies have identified many factors that affect gloss constancy, including environmental factors such as illumination (Adams, Kucukoglu, Landy, & Mantiuk, 2018; Fleming et al., 2003; Ged, Rabal-Almazor, Himbert, & Obein, 2020; Ho., Landy, & Maloney, 2006; Morimoto et al., 2023; Motoyoshi & Matoba, 2012; Olkkonen & Brainard, 2011; Pont & te Pas, 2006; Wendt & Faul, 2017), viewpoint (Ho, Maloney, & Landy, 2007), and intrinsic surface factors such as shape (Berzhanskaya, Swaminathan, Beck, & Mingolla, 2005; Morimoto et al., 2023; Nishida & Shinya, 1998; Olkkonen & Brainard, 2011; Tiedemann, 2018), diffuse reflectance (Morimoto et al., 2023; Vladusich, 2013; Wendt, Faul, Ekroll, & Mausfeld, 2010; Wendt & Faul, 2018), and surrounding context (Hansmann-Roth et al., 2017; Hansmann-Roth & Mamassian, 2017). Our approach to investigating these factors was, like most previous studies, to sample a rather arbitrary selection of shapes and illuminations and a limited range of view angles. A more thorough and systematic exploration of the impact of shape, lighting and viewpoint would be beneficial. This is challenging as the space of possible shapes and illuminations is practically infinite. One approach would be to consider parametric spaces of shape or lighting (Mazzarella, Cholewiak, Phillips, & Fleming, 2014; Mury et al., 2009; Norman, Todd, & Phillips, 2020; Ramamoorthi & Hanrahan, 2001), for example using spherical harmonics decompositions of light fields. Spherical harmonic coefficients obtained from our HDRI environment maps showed some correlation with human gloss rankings, but a regression model using these coefficients had weak predictive power and poor generalizability (for details, see Spherical Harmonics Analysis in the Supplementary Information). This limitation likely arises because, unlike the other image metrics that varied with the combination of lighting, shape, and viewpoint, spherical harmonics coefficients only capture lighting differences, reducing their predictive power.

Additionally, gloss constancy is affected by how surfaces are presented to participants; for example, studies have demonstrated that the presence of dynamic motion (Doerschner et al., 2011; Ferwerda & Padhye, 2021; Shiwen, Morimoto, Harris, & Smithson, 2023; Wendt et al., 2010; Wendt & Faul, 2018), Fresnel effects (Faul, 2019, Faul, 2021), disparity (Wendt et al., 2010), dynamic range (Doerschner, Maloney, & Boyaci, 2010), and the particular tone mapping operator used in the rendering process (Adams et al., 2018) are also important factors. We also note that previous work has shown that different image cues can be more or less predictive of human gloss perception depending on task demands, and that highlight coverage in particular is more predictive when estimating overall gloss level across a set of surfaces (e.g., see Marlow & Anderson, 2013; Qi et al., 2014). A likely reason that contrast emerged as the dominant cue in our data, while coverage underperformed relative to prior studies, is that our task required observers to judge gloss differences between image pairs rather than absolute gloss levels. How task demands affect the relative contribution of different gloss cues also deserves consideration, and future work could explore the extent to which these factors impact the predictions of HDR-VDP-3, following a similar experimental framework.

Rather than assessing how viewing conditions affect the perception of multiple reflectance differences, as is typically done in studies of gloss constancy, here we assessed how viewing conditions affect the visibility of a single, fixed difference in reflectance. This approach ensured that the observed differences in gloss perception were not confounded by variations in physical reflectance. Marlow and Anderson (2013) took a similar approach, manipulating surface geometry and the structure of the light field to assess their relative contributions to perceived gloss for a single value of physical reflectance. However, future work should test the assumption that changes in a reflectance parameter lead to proportional changes in the detectability of image differences (as predicted by HDR-VDP-3). It could be that, for some reflectance characteristics, there is a nonlinear relationship between changes in the parameter value and changes in the image. The key assumption here is that for small changes, that is, close to the JND, image changes are approximately linearly related to the reflectance characteristics. Although this assumption seems reasonable, it should be tested. Moreover, although we have shown that there is a systematic relationship between HDR-VDP-3 and gloss discrimination, this falls short of explicitly estimating a specific value for the JND from the image difference metric. Additional work is necessary to identify quantitative mappings from HDR-VDP-3 to variations in reflectance parameters, so that the JND can be expressed in terms of units of change of the reflectance parameter. Another important limitation of our study is that it considers discrimination across distinct images of the same object. For many practical applications, however, the key question is whether two juxtaposed surfaces (e.g., two doors of a car), or two neighboring parts of the same surface have the same appearance. Wendt and Faul (2019) investigated this issue by measuring detection thresholds for spatially-varying gloss within a single surface. They found that observers can be highly sensitive to abrupt spatial changes in reflectance, particularly when differences in surface smoothness occur across sharp boundaries on flat surfaces (see their Figure 12). Importantly, they also note that differences in luminance between separate surfaces—such as those used in our paradigm—may be interpreted as differences in illumination or shading rather than material, especially under static viewing conditions. This suggests that perceptual thresholds may depend not only on the magnitude of reflectance differences, but also on how those differences are spatially configured within the scene. Further research is therefore needed to clarify how sensitivity to differences in surface reflectance generalizes across different types of surface comparisons.

Finally, in the long run, it will also be necessary to generalize our approach to asymmetric comparisons (perhaps involving dynamic scenes and physical surfaces), where the difference in reflectance is confounded by differences in viewing conditions or surface color. Under these conditions, it is clear that mere image difference metrics will not capture differences in surface appearance. An image-computable model that can evaluate visual equivalence would be a useful starting point for overcoming this limitation (e.g., see Ramanarayanan, Ferwerda, Walter, & Bala, 2007). Our recent work (Morimoto et al., 2023) explored how object shape and lighting environment impacted the ability to make asymmetric comparisons of gloss across different lighting conditions and object shapes. Although substantial failures of gloss constancy were found in these experiments, participants were highly consistent in their deviations from physical ground truth. This finding agrees with the high inter-participant correlation obtained in Experiment 1 of the current study, which was also conducted online in uncontrolled viewing conditions. Apparently, whether the comparisons are symmetric or asymmetric, participants have little trouble consistently judging differences in gloss, or in making consistent adjustments to match gloss levels under different viewing conditions. The current study contributes to a growing body of literature on gloss perception, demonstrating the remarkable consistency of judgments across various viewing conditions. This means that there are good grounds for thinking that a quantitative, image-based approach can be used to predict the discriminability of gloss and other surface reflectance characteristics.

## Conclusions

Our study demonstrates the potential of using image metrics to predict gloss discrimination across a range of viewing conditions, challenging prior assumptions about the complexity of this task. Although our findings show that judgments of gloss can vary under different viewing conditions, they also reveal a surprising degree of precision in how these judgments are made. These insights not only advance our understanding of material appearance, but also point to potential practical applications in industrial quality control and computer graphics.

## Supplementary Material

Supplement 1
