# Supplementary material for: Gloss discrimination: Toward an image-based perceptual model
Source: J Vis. 2025 Aug 11;25(10):6. doi: 10.1167/jov.25.10.6 (PMC12352513; doi:10.1167/jov.25.10.6)
Supplement: Supplement 1 [file jovi-25-10-6_s001.pdf]

Supplementary Information

Spherical Harmonics Analysis

To explore the relationship between scene lighting and human gloss perception, we attempted to predict human gloss rankings using spherical harmonic (SH) coefficients extracted from the HDRI environment maps. We computed SH coefficients up to order 3 for each HDRI and examined their correlation with human gloss rankings. A subset of coefficients showed significant correlations ($r > 0.25$, $p < 0.05$). However, when these coefficients were used in a linear regression model, the predictive power was weak ($R^2 = 0.228$, *adj.* $R^2 = 0.150$), and cross-validation yielded inconsistent results (mean $R^2 = -0.061 \pm 0.243$), suggesting poor generalizability. Further, high multicollinearity among SH terms indicated that they may not independently explain perceptual gloss differences. The full coefficient output is provided in Table S1.

Coefficient	Estimate	Std Err	t-value	P>|t|	95% CI Lower	95% CI Upper
Intercept	0.0100	0.0005	21.7047	0.0000	0.0091	0.0109
SH_0_0	-0.0007	0.0026	-0.2541	0.8000	-0.0058	0.0045
SH_1_-1	0.0025	0.0025	0.9852	0.3272	-0.0025	0.0075
SH_2_1	0.0008	0.0009	0.8417	0.4022	-0.0010	0.0025
SH_3_-2	0.0009	0.0016	0.5479	0.5851	-0.0023	0.0040
SH_3_2	-0.0022	0.0010	-2.1196	0.0368	-0.0043	-0.0001
SH_3_3	0.0007	0.0018	0.4110	0.6820	-0.0029	0.0044
SH_3_5	-0.0011	0.0020	-0.5775	0.5650	-0.0050	0.0028
SH_3_10	0.0023	0.0023	1.0047	0.3178	-0.0022	0.0068
SH_3_15	-0.0001	0.0009	-0.1074	0.9147	-0.0019	0.0017
SH_3_20	-0.0009	0.0011	-0.7842	0.4350	-0.0030	0.0013
SH_3_21	-0.0022	0.0022	-0.9853	0.3271	-0.0065	0.0022
SH_3_22	-0.0025	0.0014	-1.7318	0.0867	-0.0053	0.0004
SH_3_27	-0.0002	0.0013	-0.1311	0.8960	-0.0028	0.0024
SH_3_28	0.0011	0.0010	1.1693	0.2454	-0.0008	0.0031
SH_3_29	-0.0005	0.0011	-0.4717	0.6383	-0.0027	0.0017
SH_3_32	0.0026	0.0019	1.3336	0.1857	-0.0013	0.0064

Table S1. Spherical Harmonic coefficients included in the ordinary least squares model.

Object Size Analysis

To test the possibility that object size contributed to model predictions, we computed the mean of the HDR-VDP-3 probability map (P_{map}) in two ways: (1) across all image pixels (our original metric), and (2) restricted to pixels belonging to the object, labeled in the alpha channel of the HDR image files. Both metrics were then correlated with the human judgments collected in Experiment 1 (panels A and B in Figure S1). Note that this object-only version of the metric explains an additional 10 percentage points of variance (R^2 from .66 to .76 for the full-image and object-only metric, respectively). In addition, we computed the proportion of image pixels occupied by the object (proximal object size) and tested its correlation with the original model scores (panel C). While the object-restricted mean P_{map} slightly improved the correlation with human data, object size itself showed only a weak, non-significant correlation with the original P_{map} scores (r = 0.17, p = 0.08). This suggests that proximal object size alone does not account for the model's predictive power.

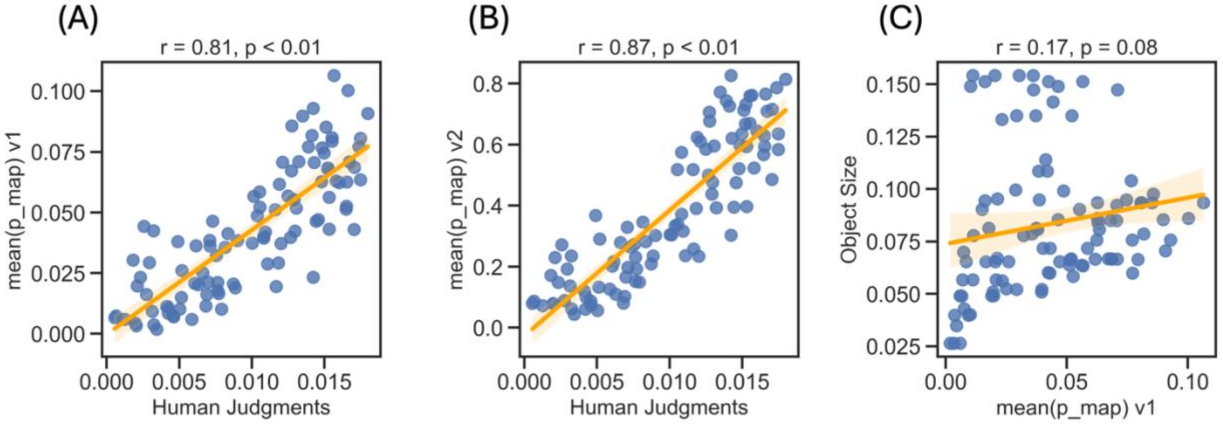

Figure S1. Correlation between human judgments and (A) the original mean P_{map} computed over the entire image, (B) the mean P_{map} computed only over object pixels, and (C) the relationship between object size (as a proportion of image pixels) and the original mean P_{map} metric.
